# Prevalence of ocular *Chlamydia trachomatis* infection and antibodies within districts persistently endemic for trachoma, Amhara, Ethiopia

**DOI:** 10.1371/journal.pntd.0012900

**Published:** 2025-03-11

**Authors:** Mary K. Lynn, Zebene Ayele, Ambahun Chernet, E. Brook Goodhew, Karana Wickens, Eshetu Sata, Andrew W. Nute, Sarah Gwyn, Nishanth Parameswaran, Demelash Gessese, Mulat Zerihun, Kimberly A. Jensen, Gizachew Yismaw, Taye Zeru, Adisu Abebe Dawed, Fikre Seife, Zerihun Tadesse, E. Kelly Callahan, Diana L. Martin, Scott D. Nash

**Affiliations:** 1 The Carter Center, Trachoma Control Program, Atlanta, GeorgiaUnited States of America; 2 University of South Carolina, Columbia, South Carolina, United States of America; 3 The Carter Center, Trachoma Control Program, Addis Ababa, Ethiopia; 4 U.S. Centers for Disease Control and Prevention, Atlanta, GeorgiaUnited States of America; 5 Synergy America, Inc., Duluth, GeorgiaUnited States of America; 6 Amhara Public Health Institute, Research and Technology Transfer Directorate, Bahir Dar, Ethiopia; 7 Amhara Regional Health Bureau, Health Promotion and Disease Prevention, Bahir Dar, Ethiopia; 8 Ethiopia Ministry of Health, Disease Prevention and Control Directorate, Addis Ababa, Ethiopia; Medical University of Vienna, AUSTRIA

## Abstract

**Background:**

Persistent trachoma is increasingly recognized as a serious concern for the global trachoma program. Persistent trachoma is defined as those districts that have had two or more trachoma impact surveys in which the trachomatous inflammation—follicular (TF) prevalence has never been <5%, the elimination threshold for TF. Enhanced tools such as infection and serological monitoring elucidate long-term transmission patterns within persistent districts. This study aimed to clarify trachoma intensity via both traditional indicators and *Chlamydia trachomatis* (*Ct*) infection and serologic markers in four districts experiencing persistent trachoma with >10 years of interventions.

**Methodology:**

Population-based surveys were conducted in 2019 in four trachoma persistent districts. Children ages 1-9 years were examined for trachoma clinical signs and children 1-5 years were swabbed for *Ct* infection. Antibodies to the trachoma antigens Pgp3 and CT694 were measured for all individuals ≥1 year, assessed by multiplex bead assay. Seroconversion rates (SCRs) to both antigens were estimated for children and for individuals of all ages.

**Results:**

One district, Ebinat, remained highly endemic, with a TF prevalence and infection prevalence (ages 1–5 years) of 42.5% and 7.1% respectively. Indicators were lower in the other three districts ranging from 10.7%-17.9% TF and 0%-1.7% infection. The Pgp3 SCR among children ages 1–9 years was considerably higher in Ebinat with 10.8 seroconversions per 100 child-years, (95% Confidence Interval [CI]: 8.2, 14.4) compared to the other three districts (SCR range: 0.9–3.9). All-age Pgp3 SCR estimates detected a significant decline in seroprevalence in Machakel district at approximately 12 years prior to 2019.

**Conclusions:**

Infection and serology may be useful tools for clarifying transmission, particularly among persistent districts, and ongoing interventions likely helped push these hyperendemic districts towards the elimination threshold. However, districts such as Ebinat may require more intense interventions to reach elimination within acceptable timelines.

## Author summary

Persistent trachoma is increasingly recognized as a serious concern for the global trachoma program. Persistent trachoma is defined as those districts that have had two or more trachoma impact surveys in which the trachomatous inflammation—follicular (TF) prevalence has never been less than 5%, the elimination threshold for the TF indicator. Enhanced tools such as infection and serological monitoring can elucidate long-term transmission patterns within persistent districts. This study aimed to clarify trachoma intensity via both traditional indicators (TF) and *Chlamydia trachomatis* infection and serologic markers in four districts experiencing persistent trachoma. Surveys were conducted in these four districts in 2019 after more than 10 years of interventions. Although all districts had a TF prevalence greater than 5%, one remained highly endemic, with a high prevalence of TF, infection, and antibodies. Trachoma indicators were lower in the other three districts. Serological patterns across the age-span detected a significant decline in seroprevalence in one district approximately 12 years prior to the survey, coinciding with the beginning of trachoma interventions. Infection and serology may be useful tools for clarifying transmission, particularly among persistent districts. However, some districts may require more intense interventions to reach trachoma elimination within acceptable timelines.

## Introduction

*Chlamydia trachomatis* (*Ct*) is the etiologic agent for trachoma, the leading infectious cause of blindness [1]. The debilitating ocular outcomes attributable to trachoma develop over time, after repeated infections with *Ct*. The World Health Organization (WHO) has endorsed the Surgery, Antibiotics, Facial Cleanliness, and Environmental Improvement (SAFE) strategy to eliminate trachoma as a public health problem globally. Depending on the baseline prevalence of trachomatous inflammation—follicular (TF) among children ages 1 to 9 years, control programs deliver 1 to 7 years of SAFE interventions followed by trachoma impact surveys (TIS) to measure impact and progress towards trachoma elimination [1]. While the global elimination of trachoma by 2020 was not achieved as targeted, the global program has achieved a 90% reduction in the number of individuals at risk for trachoma-induced blindness since 1996 [1,2].

One challenge to achieving the global elimination of trachoma is that some districts, particularly in Ethiopia, are experiencing persistent trachoma, defined as having had two or more TIS in which the TF prevalence has never been <5%, the elimination threshold for the TF indicator [3]. In endemic regions such as Amhara, Ethiopia, this definition captures a wide range of districts from those close to 5% to those still hyperendemic (defined programmatically as TF ≥30%) after two or more TIS [4]. Among those districts in the latter category, ongoing transmission is a clear threat to ocular health and to Ethiopia achieving the elimination of trachoma as a public health problem by the new global target of 2030. Given the disparate trajectories of TF prevalence observed across districts in Amhara, it is likely that not all districts with persistent trachoma are epidemiologically similar. To better understand persistent trachoma, experts have called for increased monitoring strategies such as collecting *Ct* infection and serological data alongside clinical data to better characterize transmission in these settings [3].

During the TIS conducted in 2019 in Amhara, complementary indicators including *Ct* infection and antibodies to *Ct* antigens were collected alongside routine data. Four districts experiencing persistent trachoma were chosen for enhanced monitoring and evaluation due to substantial variation observed in the TF indicator across the districts. This study aimed to clarify *Ct* transmission dynamics, both current and historical, in these districts which have received over 10 years of SAFE interventions yet remain persistently trachoma endemic.

## Methods

### Ethics statement

This study was approved by the Amhara Regional Health Bureau and by the Institutional Review Board (IRB) at Emory University (under protocol 079-2006). The protocol for the 2019 survey was further reviewed by Tropical Data staff (https://www.tropicaldata.org/) which ensures adherence to the WHO standardized approach for TIS [5]. Informed verbal consent was obtained and recorded from all heads of households, study participants, and parents of participating children due to the extent of illiteracy in the region. Verbal assent was also obtained from participating minors in accordance with the Declaration of Helsinki. Participants were allowed to withdraw consent at any time for any reason. Staff at the U.S. Centers for Disease Control and Prevention (CDC) were not engaged in research on human subjects, did not have access to personal identifiers, and did not have contact with any study participants.

### Setting and timeline

In August 2019, population-based surveys were conducted in four districts in Amhara experiencing persistent trachoma. These districts are non-contiguously located in central Amhara, within the South Gondar (Ebinat) and East Gojam (Debay Tilatgin, Goncha, and Machakel) zones (S1 Fig). The pre-intervention baseline TF prevalence in Ebinat district (2001) and Goncha district (2003) were 79% and 87% respectively (S2 Fig) [6]. Based on these results, SAFE interventions began in these districts, and they received two TIS over time to measure impact. The most recent TF prevalence estimates from TIS in Ebinat (2014) and Goncha (2016) were 49.5% and 24.0% respectively [4,7]. Debay Tilatgin district and Machakel district in East Gojam zone were surveyed in 2007 as part of a pre-intervention zonal-level baseline survey which determined that the prevalence in that zone was 48.3% [8]. SAFE interventions began in these two districts, and they each received district level TIS in 2013 and again in 2016. The TF prevalence in 2016 was 27.9% in Debay Tilatgin and was 14.1% in Machakel. These districts were among those due for a TIS in 2019 and were selected purposely as they had received >10 years of SAFE implementation and represented a range of TF prevalence (14.1%-49.5%) among districts considered persistent. These districts received their last MDA 6 (Ebinat) and 8 (the remaining three districts) months prior to these surveys.

Since 2009, mass drug administration (MDA) administrative coverage as part of the SAFE strategy has exceeded 80% in all four districts (S3 Fig). This level of coverage has been supported by population-based coverage surveys [9,10]. Facial cleanliness and environmental improvement interventions in Amhara over this time-period included a school trachoma program which provided health and hygiene education to over 8,000 primary schools in the region [4,7,11]. Community education was conducted via the MDA program and through the employment of hygiene and environmental health officers. The advocacy for latrine construction, a critical component of the environmental improvement of SAFE strategy, was further conducted. An average of 500,000 latrines have been built each year, leading to considerable increases in latrine coverage since baseline surveys were conducted [4,7].

### Survey design

Multi-stage cluster randomized sampling was used to select 30 communities using a population proportional to estimated size approach. Within each community, one segment, (a development team, an administrative unit in Ethiopia comprised of approximately 30 households) was then randomly selected by a community representative [4]. All household residents ≥1 year of age from selected segments were enumerated and all consenting members were examined for trachoma clinical signs and provided a dried blood spot (DBS) sample, and children ages 1 to 5 years provided a conjunctival swab.

Sample size estimates were based on assumptions of 4% TF ± 2% and a 2.63 design effect to ultimately a target sample size of 1,164 children in each district [4,5]. Any individual diagnosed with TF or trachomatous inflammation—intense (TI) was offered treatment according to WHO guidelines, and individuals diagnosed with trachomatous trichiasis (TT) were referred for surgery. Demographic and household questionnaires were administered to heads of households using Tropical Data software on cellular phones, and included questions regarding water availability, presence of latrines, and sanitation and hygiene practices. Further details regarding the sample design have been previously published [4,5,7].

### Training and clinical examination

A seven-day classroom and field-based training for grading certification was conducted prior to the surveys. At the end of the training, a field-reliability exam was conducted wherein trainees graded 50 conjunctivae from children ages 1 to 9 years, and their grades were compared via Kappa method to one previously certified “grader trainer.” Certification was awarded to those trainees scoring a Kappa statistic ≥0.7 [4,5]. Re-certification was required for returning graders prior to the onset of each survey round. Certified graders examined survey participants for TF, TI, and TT using a binocular loupe with 2.5x magnification and sufficient lighting.

### Sample collection

Graders collected conjunctival swabs from all examined and assenting children ages 1 to 5 years using polyester-tipped swabs (Fisher Scientific, Waltham, MA, USA) with appropriate aseptic technique and personal protective equipment. Graders firmly swabbed the upper tarsal conjunctiva three times, rotating the swab 120˚ along the axis at each pass to ensure sufficient epithelium capture [12]. Swabs were placed into labeled 2.0 mL cryovials and transported in vaccine coolers at approximately 4˚C. Samples were stored at -20˚C until the time of assay. For quality control, “air swabs” were also collected from approximately 5% of sampled children by passing a dry, sterile swab within one inch of the conjunctiva.

DBS were collected from all consenting participants by a trained laboratory technician via retractable lancet and filter paper (TropBio Pty Ltd., Townsville, Queensland) [13]. Approximately 10 µL of finger prick blood was taken for each of the six blood spot extensions (6 mm each) contained on the filter paper. Labeled filter paper was air-dried for a minimum of two hours, placed in a sealed plastic bag with desiccant, and transported for storage at -20˚C until shipped to the CDC in Atlanta, Georgia, USA.

### Laboratory processing

Conjunctival swabs were processed for each district in randomized pools of five individual samples to each pool at the Trachoma Molecular Laboratory at the Amhara Public Health Institute in Bahir Dar, Ethiopia. Laboratory technicians were masked to the clinical sign status of each sample, the originating district of pools, and whether samples were air swabs or from participants. Air swabs were only pooled with other air swabs and were assayed on the same plates as participant pools. Pools were assayed by Real*Time* polymerase chain reaction (PCR) using the Abbott m2000 Real*Time* system (Abbott Molecular Inc., Des Plaines, IL, USA) in February 2020. Rigorous quality control measures were maintained throughout all phases of sample processing as previously described [14].

Sample preparation and detection of anti-*Ct* antibodies were conducted by multiplex bead assay (MBA) at the CDC as previously described [15]. Briefly, DBS extensions were eluted overnight at 4°C and diluted to a final sera concentration of 1:400 in Buffer B (1X phosphate buffered saline (PBS), 0.5% casein, 0.5% polyvinyl alcohol, 0.8% polyvinylpyrrolidone, 0.3% Tween 20, 0.02% NaN_3_) containing 3 µg/mL *Escherichia coli* extract.

Beads coupled with *Ct* antigens Pgp3 and CT694 were incubated with diluted sample in 96-well filter bottom plates (Millipore, Bedford, MA) for 1.5 hours then washed with 0.05% Tween-20 in PBS (PBST). Beads were incubated for 45 minutes with biotinylated mouse anti-human total IgG (Clone H2; Southern Biotech, Birmingham, AL) and biotinylated mouse anti-human IgG4 (clone HP6025; Southern Biotech) to detect total IgG anti-*Ct* antibodies bound to antigen on the beads. After additional washes, beads were incubated for 30 minutes with streptavidin-phycoerythrin (SAPE Invitrogen, South San Francisco, CA) to detect bound biotinylated anti-human IgG. Loosely bound antibodies were removed after detection by a final 30-minute wash with PBST containing 0.5% BSA and 0.02% NaN3. After washing, beads were resuspended in PBS and stored overnight at 4°C.

The next day, plates were read on a Bio-Plex 200 instrument (Bio-Rad, Hercules, CA) equipped with Bio-Plex manager 6.0 software (Bio-Rad). The median fluorescence intensity (MFI) with background (BG) signal (Buffer B alone) subtracted out (MFI-BG) was recorded for each antigen for each sample. A receiver-operating characteristic curve panel based on previously identified positive and negative sera was used to determine MFI-BG positivity thresholds of 783 (Pgp3) and 102 MFI-BG (CT694).

### Data analysis

District-level prevalence estimates for TF were provided by Tropical Data service and were calculated using the mean of the age-adjusted (1-year age bands using the Ethiopian National census) cluster level data. A previously described bootstrapping method was used to obtain confidence intervals (CI) for district estimates [4,16]. District prevalence estimates and CI for TI, clean face, and antibodies were also calculated using this method. *Ct* infection was defined as any detectable *Ct* bacterial DNA detected in a pool. District infection prevalence for each district was estimated from the pooled prevalence as the number of individual samples most likely to have resulted in the pooled results [12,17]. Only point estimates are reported as this estimation procedure does not allow for the calculation of confidence intervals around the point estimate. Map was created in ArcGIS Pro 2.2.6 (ESRI, Redlands, CA) using shapefiles sourced from the GADM database (gadm.org).

Outcomes for serology assays were described both as binary and continuous variables using the previously described positivity threshold. Seroconversion rates (SCR) to antigens Pgp3 and CT694 were obtained by generalized linear model with complementary log-log link and robust standard errors to estimate *Ct* force of infection among children (ages 1 to 9 years) per child-year [13,18,19]. The model assumed constant force of infection and no seroreversion. SCRs were estimated for children ages 1 to 9 years from age-structured seroprevalence and for all ages separately. Results for this analysis are reported as seroconversions per 100 children per year for ease of interpretation.

SCRs were estimated for all ages using more complex models than those employed for estimating SCRs among children ages 1 to 9 years. For all ages, we estimated SCRs using two distinct sero-catalytic models as described in previous works [20,21]. Briefly, both models use a Bayesian Markov chain Monte Carlo method to estimate seroconversion and seroreversion rates using age as a proxy for time. Model 1 assumed a constant age-dependent rate of seroconversion and seroreversion. Model 2 assumed a time point of change in which the seroconversion rate significantly and substantially decreased. Model 2 estimates a historic seroconversion rate prior to this time point of change, the proportional decline in transmission, seroreversion rate, and seroconversion rate after the time point of change*.* All-age model diagnostics were compared for each antigen and district via the fit plots of autocorrelation, deviance information criteria (DIC), Gelman-Rubin statistic (GR), and effective sample size (ESS) to determine the best fitting model. Previously published informative priors for the proportional decline in transmission and seroreversion rate were additionally applied to Model 2 for all districts excluding Machakel [20]. The best fitting models for each district are presented below. All statistical analyses were performed in R Studio version 4.1.1 (RStudio, PBC, Boston, MA) with the packages “mgcv”, “sandwich”, “lmtest”, “coda”, “binom”, and “mass”.

## Results

Among the 3,139 children ages 1 to 9 years enumerated in the four surveyed districts, 3,065 (97.6%) were examined for trachoma clinical signs (Table 1). A total of 1,708 children ages 1 to 5 years were swabbed for *Ct* infection. DBS were collected from 2,695 children ages 1 to 9 years and from 7,738 individuals ages ≥10 years. From the household Water, Sanitation, and Hygiene (WASH) questionnaire, household latrine presence was 19.0% in Ebinat and >50.0% in the remaining districts (S1 Table). The prevalence of household access to water within 30 minutes ranged from 30.4% to 42.0% and the prevalence of clean face ranged from 46.5% to 70.1% among districts.

**Table 1:** Sample size for the four district surveys in Amhara, Ethiopia, 2019.

	HH	ENU	EXD	SEROLOGY	INFECTION	ENU	EXD	SEROLOGY
**DISTRICT**	**SURVEYED**	**1-9Y**	**1-9Y**	**1-9Y**	**1-5Y**	**10** ^+^ **Y**	**10** ^+^ **Y**	**10** ^+^ **Y**
Ebinat	900	957	926	861	536	2,713	1,853	1,827
Goncha	899	727	713	670	360	2,900	2,230	2,214
Debay Tilatgin	900	555	545	506	311	2,627	2,121	2,102
Machakel	900	900	881	794	501	2,728	2,230	2,038

HH: household; ENU: individuals enumerated; EXD: individuals examined; Y: year.

The TF prevalence among children ages 1 to 9 years was highest in Ebinat at 42.5% (95% CI: 35.1, 49.9) followed by Goncha at 17.9% (95% CI: 13.4, 23.4) (Table 2). TF prevalence within this age group was similar among Debay Tilatgin at 10.8% (95% CI: 6.3, 15.8) and Machakel at 10.7% (95% CI: 7.2, 15.2). The TI prevalence was 4.3% (95% CI: 2.5, 6.5) in Ebinat, and just over 1% in the other three districts. *Ct* infection among children ages 1 to 5 years was present in three of the four districts reaching a high of 7.1% in Ebinat. No infection was detected in Machakel in this age group. No pools containing air swabs were positive.

**Table 2:** Prevalence of TF, TI, TT, antibodies to Pgp3, CT694, and *Ct* infection, Amhara, Ethiopia, 2019.

DISTRICT	TF 1-9 Y	TI 1-9 Y	TT 15^+^ Y	PGP3 1-9 Y	CT694 1-9 Y	CT 1-5 Y
Ebinat	42.5%	4.3%	2.8%	38.2%	40.9%	7.1%
	(35.1, 49.9)	(2.5, 6.5)	(2.1, 3.6)	(31.0, 46.0)	(35.1, 46.8)	
Goncha	17.9%	1.3%	4.3%	15.9%	19.2%	1.7%
	(13.4, 23.4)	(0.5, 2.3)	(3.3, 5.3)	(11.4, 20.5)	(14.4, 24.9)	
Debay Tilatgin	10.8%	1.1%	1.3%	11.0%	13.9%	1.6%
	(6.3, 15.8)	(0.3, 2.2)	(0.7, 1.8)	(6.4, 16.0)	(8.8, 19.3)	
Machakel	10.7%	1.1%	2.4%	5.7%	9.4%	0.0%
	(7.2, 15.2)	(0.1, 2.7)	(1.6, 3.3)	(2.6, 9.5)	(6.3, 13.3)	

TF: trachomatous inflammation-follicular; TI: trachomatous inflammation-intense; TT: trachomatous trichiasis; CT: Chlamydia trachomatis; Y: year; Pgp3 and CT694: refer to trachoma antigens. Numbers in parentheses are 95% confidence intervals. For CT estimates, estimation procedures used do not allow for the calculation of CI around the point estimate.

**Fig 1:**
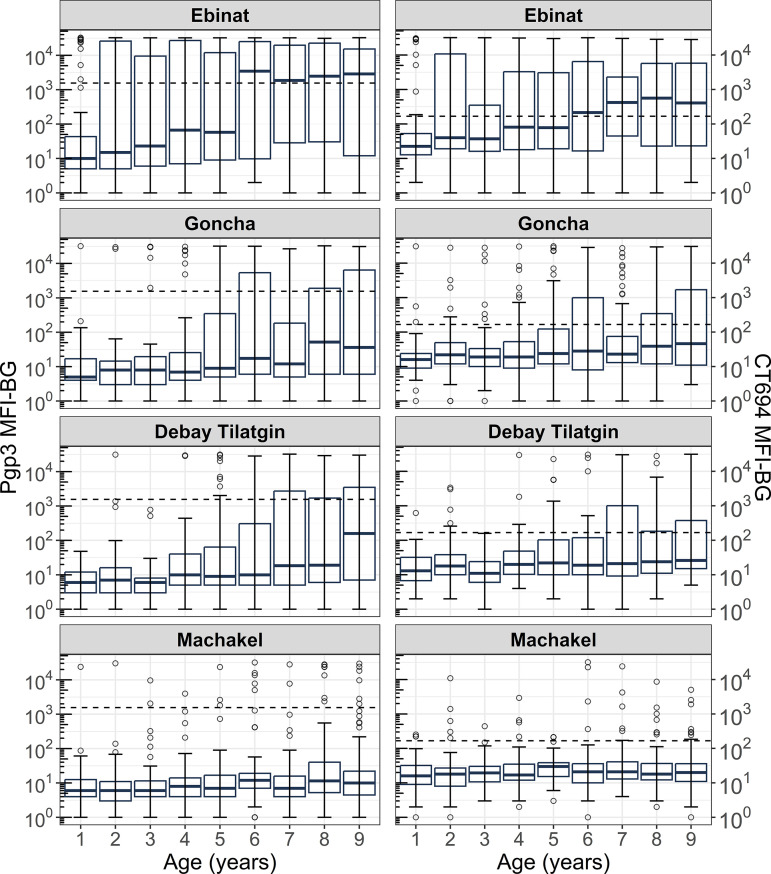
Distribution of antibodies (log MFI-BG) by year of age, Amhara, Ethiopia, 2019. Age-specific antibody distribution is shown for children 1 to 9 years using a box plot. The upper and lower bounds of each box represent the first and third quartiles of the interquartile range, respectively. The horizontal line within each box represents the median. The vertical lines in the center of each box connect the minimum and maximum values. Circles represent individual log MFI-BG outliers.

**Fig 2:**
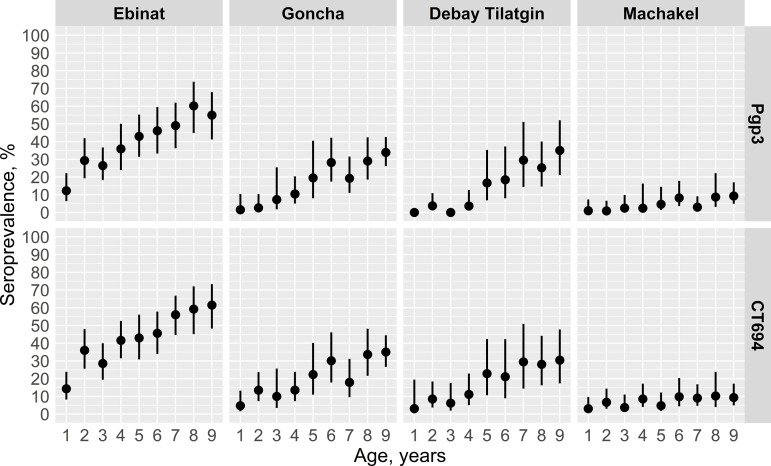
Age-specific seroprevalence among children ages 1 to 9 years by district, Amhara, Ethiopia, 2019. Estimated age-specific prevalence of antibodies is shown for trachoma antigens Pgp3 and CT694. Points represent estimated seroprevalence by year of age and lines represent estimated 95% confidence intervals.

Antibodies to both antigens, measured by MFI, were consistently highest in Ebinat across ages 1 to 9 years compared to other districts (Fig 1). Age-specific seroprevalence across this age range followed a similar pattern (Fig 2). The age-group prevalence of antibodies to Pgp3 and CT694 among children 1 to 9 was also highest in Ebinat at 38.2% (95% CI: 31.0, 46.0) and 40.9% (95% CI: 35.1, 46.8), respectively (Table 2). Seroprevalence of Pgp3 within this age group in the other three districts ranged from 5.7% (95% CI: 2.6, 9.5) in Machakel to 15.9% (95% CI: 11.4, 20.5) in Goncha, while the seroprevalence of CT694 ranged from 9.4% (95% CI: 6.3, 13.3) in Machakel to 19.2% (95% CI: 14.4, 24.9) in Goncha.

**Fig 3:**
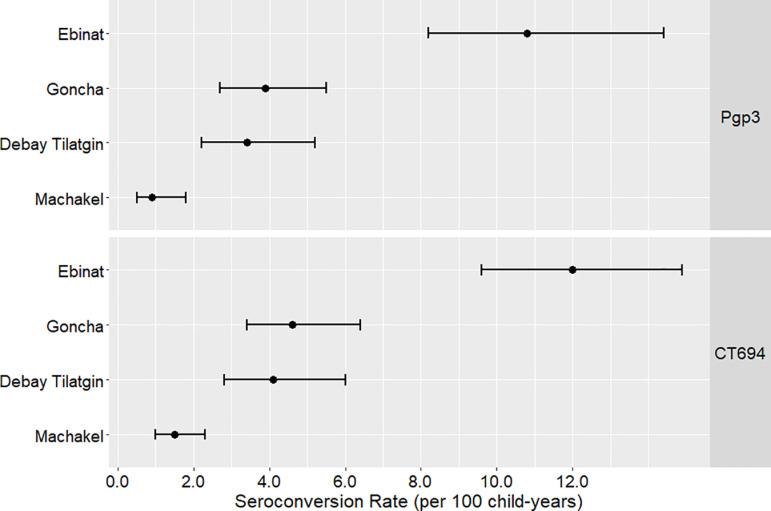
Estimated seroconversion rates among ages children 1 to 9 years, Amhara, Ethiopia, 2019. Points represent estimated seroconversion rates (per 100 person-years) and lines represent estimated 95% confidence intervals for the 1 to 9 years age group by district.

In Ebinat, the Pgp3 SCR among children ages 1 to 9 years was 10.8 (95% CI: 8.2, 14.4) seroconversions per 100 child years (Fig 3). The SCR for Pgp3 in Goncha (3.9, 95% CI: 2.7, 5.5) and Debay Tilatgin (3.4, 95% CI: 2.2, 5.2) were similar, while Machakel had the lowest of the four districts (0.9, 95% CI: 0.5, 1.8). Similar trends were observed for CT694, with the SCRs ranging from 1.5 seroconversion per 100 child years in Machakel (95% CI: 1.0, 2.3) to 12.0 seroconversions per 100 child years in Ebinat (95% CI:9.6, 14.9).

**Fig 4:**
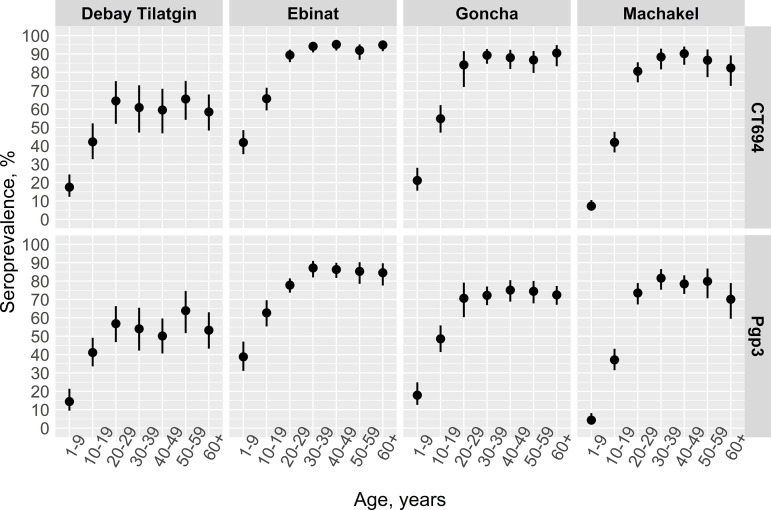
Age-specific seroprevalence among individuals of all ages by ten-year age groups, Amhara, Ethiopia, 2019. Seroprevalence, or antibodies, are shown for the trachoma antigens Pgp3 and CT694. Points represent average estimated seroprevalence of ten-year age groups and lines represent estimated 95% confidence intervals.

Seroprevalence across the whole population was again highest in Ebinat for nearly all age groups compared to the other three districts (Figs 4 and S4). Diagnostics from the best fitting all-age serocatalytic model indicated Model 1, a constant age-dependent rate of seroconversion, was best fitting to the data from Ebinat (Tables 3, S2 and S5 and S6 Figs). For Machakel, Model 2 was the best fit, which assumed a time point of change in which the seroconversion rate decreased. The SCR for Pgp3 declined significantly 12.3 years (Inter quantile range [IQR] 2.5%, 97.5%: 11.4, 13.0) prior to the survey. Prior to the time point of change, SCR in Machakel was 27.5 (95% Credible interval [CrI]: 20.6, 39.0) seroconversions per 100 person-years, decreasing to 1.1 (95% CrI: 0.8, 1.4) seroconversions per 100 person-years after this point. Similar SCRs were observed for the antigen CT694 in this district. Model fit varied by antigen in Goncha and Debay Tilatgin. In both districts for the antigen Pgp3, Model 2 was selected with the inclusion of informative priors. For CT694, significant autocorrelation was observed in Model 2 for Goncha, and Model 2 did not converge in Debay Tilatgin. Therefore Model 1 was the best fit for CT694 in these districts.

**Table 3:** Estimated SCR for children 1-9 and for all ages with 95% CI, Amhara, Ethiopia, 2019.

				SCR 1 ALL AGE	SCR 2 ALL AGE	TIME POINT OF CHANGE	
		STATIC SCR 1-9 YEARS*	MODEL	BEFORE POINT OF CHANGE	AFTER POINT OF CHANGE	YEARS AGO	
DISTRICT	ANTIGEN	(2.5%, 97.5% IQR)	(1 OR 2) ˥	(2.5%, 97.5% IRQ)	(2.5%, 97.5% IRQ)	(2.5%, 97.5% IQR)	DIC
Ebinat	Pgp3	10.8 (8.2, 14.4)	1	10.1 (9.1, 11.3)^*^	--	--	2921.1
	CT694	12.0 (9.6, 14.9)	1	10.4 (9.6, 11.3)^*^	--	--	2469.8
Goncha	Pgp3	3.9 (2.7, 5.5)	2	7.8 (6.7,9.1)	0.6 (0.2,1.1)	2.5 (1.6,3.5)	3289.5
	CT694	4.6 (3.4, 6.4)	1	6.3 (5.8, 6.8)^*^	--	--	2800.1
Debay Tilatgin	Pgp3	3.4 (2.2, 5.2)	2	9.1 (6.9,12.8)	1.1 (0.3,2.7)	3.7 (2.7,5.4)	3212.0
	CT694	4.1 (2.8, 6.0)	1	5.3 (4.7, 6.0)^*^	--	--	3234.0
Machakel	Pgp3	0.9 (0.5, 1.8)	2	27.5 (20.6, 39.0)	1.1 (0.8, 1.4)	12.3 (11.7, 12.8)	2477.0
	CT694	1.5 (1.0, 2.3)	2	27.9 (20.5, 38.2)	1.5 (1.2, 1.9)	12.4 (11.4, 13.0)	42273.7

All estimates of SCR presented in the table represent SCR per 100 person-years. Static estimates of SCR are shown for children ages 1-9 years for both antigens in all districts. Among all age estimates, static SCR was reported for all districts and antigens where Model 1 was a superior fit, as a time point of change cannot be identified by this model, denoted by *. All ages SCR estimates for which model was a better fit show estimated SCR before and after the identified time point of change. ˥: Model 1 serocatalytic model assuming steady seroconversion rate; Model 2: serocatalytic model assuming a significant time point of change in all age seroconversion rate; SCR: seroconversion rate, IQR: interquartile range, DIC: deviance information criteria, CI: 95% confidence interval.

## Discussion

Though all four districts in this study would still be considered trachoma persistent as of these surveys, infection and seroprevalence data including SCRs between districts demonstrate distinct epidemiologic patterns. As of 2019, Ebinat remained hyperendemic (TF >30%) with an SCR among children of 10.8 (Pgp3) seroconversions per 100 person-years and an infection prevalence of 7.1%. Transmission intensity and infection prevalence were considerably lower in the other three districts. While persistent trachoma remains a clear threat to trachoma elimination, these results demonstrate that the current definition is likely too broad to adequately describe the various transmission patterns observed in Amhara. The expansion of the use of complementary indicators within surveys conducted in persistent districts would provide useful information for monitoring trachoma control programs.

To enhance the evaluation of the SAFE strategy, the Trachoma Control Program in Amhara began monitoring *Ct* infection at scale in 2011 [12]. Data on *Ct* infection prevalence and infection load allowed for further insights into areas at high risk for continued infection [22]. Since this time, studies have revealed a poor correlation between *Ct* infection and TF prevalence in settings where multiple MDA rounds have been administered [23–25]. TF has also been shown to exceed *Ct* prevalence in post-MDA settings, presenting a need for complementary indicators of transmission [23,26]. As such, in 2017, collection of serologic data began in Amhara as a second complementary measure to assess transmission dynamics and elimination progress within districts [13,27]. The SCR provides insight into current community-level *Ct* transmission, as high SCRs among children may indicate active transmission within communities [24,28]. Modeling techniques can further be used to identify historic patterns in transmission dynamics, and Bayesian modeling across the age range can be useful for modeling in areas without longitudinal serology data [29]. Indeed, different historical trachoma trajectories were detected among these four surveyed districts. As demonstrated by the results of this study, the use of these complementary indicators in conjunction with traditional clinical trachoma measures was necessary to fully clarify trachoma transmission and progress towards reaching elimination goals.

Ebinat district may be one of the most trachoma-endemic districts remaining in the world. A baseline survey conducted in 2001 demonstrated a TF prevalence of 79%, and despite ongoing AFE interventions of the SAFE strategy since 2003, the district has not achieved a TF prevalence below 40% in any of its four TIS [6]. Results of this current study demonstrated ongoing *Ct* transmission among children in Ebinat, with a *Ct* infection prevalence of 7.1% and an SCR between 11 (Pgp3) and 12 (CT694) seroconversions per 100 child years. This places Ebinat near the top of those trachoma-endemic districts where SCRs has been calculated using comparable methodology [28]. The all-age serology data further demonstrated that a decline in seropositivity has not occurred over time in this district despite considerable intervention pressure. Elucidating the reasons behind this continued transmission in this district will be critical to inform potential programmatic improvements. While it is possible that the strains of *Ct* circulating in Ebinat may be less susceptible to azithromycin MDA pressure, studies have not found evidence of antimicrobial resistance to macrolides in ocular *Ct* strains, including those circulating in this district [30,31]. Household WASH indicators were lowest in Ebinat among the districts in this study, which included latrine presence (19.0%), access to water in less than 30 minutes (30.4%), and clean face (46.5%). Observational studies have continually demonstrated significant relationships between WASH and trachoma prevalence [32–34]. In Amhara specifically, statistically significantly less active trachoma (TF and/or TI) was observed when community sanitation coverage exceeded 80% [35]. Randomized control trials, however, have been less clear on the efficacy of this “E” component of SAFE strategy in this region [36]. Clearly, SAFE interventions in Ebinat need to be strengthened and enhanced, and this includes improving the availability of water and latrines and increasing the number of MDA rounds per year.

While Machakel is considered a trachoma-persistent district based on the TF indicator, the transmission dynamics observed in the district were considerably different than that of Ebinat. As of the current TIS, no *Ct* infection was detected among children ages 1 to 5 years, and the SCRs per 100 child years was less than 1.0 for Pgp3. All-age sero-catalytic models identified a time-point of significant decline in seropositivity (Pgp3 & CT694) approximately 12 years (IQR 2.5%, 97.5%: 11.4, 13.0) prior to the 2019 survey. Models suggested an all-age change in Pgp3 SCRs from 27.5 seroconversions per 100 individuals per year prior to the point of change to 1.1 seroconversions per 100 individuals per year after this time point of change. Both the serological data and the observed TT prevalence of 2.4%, support the fact that this district was historically highly endemic for trachoma. The identified time point of change approximately coincides with the onset of AFE interventions, including the MDA program in Machakel in 2008. These results suggest that these programmatic interventions may have significantly contributed to reductions in trachoma transmission, even though Machakel has experienced slow progress towards reaching the <5% TF elimination threshold. Despite evidence for low *Ct* transmission, including the absence of detectable *Ct* infection, the observed TF prevalence in Machakel was 10.7% in this study, and thus per WHO recommendations, this district is eligible to receive an additional three years of A, F, and E interventions. While more research is needed to determine operational thresholds for serological and infection indicators, these data suggest that these additional rounds of treatment may not be needed in Machakel [25,26,28].

Goncha and Debay Tilatgin districts both had TF >10%, with concurrent infection prevalence >1% and SCRs ranging between three and four seroconversions per 100 child years for Pgp3. While SCR thresholds for programmatic decision making have not yet been developed, compared to a previous meta-analysis, the SCRs observed in these districts ranks these two among districts considered endemic with detectable *Ct* infection [28]. Trachoma has been well characterized in Goncha specifically as it was the setting of several trachoma-focused randomized control trials [37,38]. Prior to the start of MDA in those trials, the average *Ct* infection prevalence among children was reported as 40%. Over the course of 3.5 years of high treatment coverage MDAs within the trial communities, the prevalence of *Ct* infection dropped to as low as 1.9% in the annually treated arm, a level similar to that observed here districtwide [37]. In the follow-up trial in Goncha, MDA was discontinued in a random set of the same communities, and *Ct* infection returned to a prevalence of 20% [38]. This suggests that the required infection threshold needed to prevent district-level recrudescence after MDA discontinuation is likely still lower than 1.9%. It also suggests that programmatic annual MDA has likely been keeping *Ct* infection low in Goncha and Debay Tilatgin. The results of this study suggest that MDA and other interventions have been beneficial in Goncha and Debay Tilatgin and that given the already long treatment history in these districts, more frequent than annual MDA might be warranted [3].

Serocatalytic Bayesian models have previously been used to evaluate trachoma recrudescence, primarily in post-elimination, low-endemicity settings [29,39]. The need to further understand the utility of serological monitoring in heterogeneous settings has been identified, and this study fills a gap in employing multiple seroconversion models in settings with varying levels of trachoma persistence[24]. Methods used in this study were consistent with prior works that allowed for estimation of both seroconversion and seroreversion [20,29]. This model further accounts for exposure across the age range to estimate SCRs and to clarify changes in patterns of transmission over time. Primarily these models confirmed that all four of these districts had historically high levels of *Ct* transmission. Through this approach, this study further determined that one district (Machakel) had a significant change in transmission, likely program related, while distinct transmission changes were not observed in the other three despite many years of intense interventions [4,7]. It has been well documented that seropositivity cannot disentangle exposure to ocular or urogenital Chlamydia, and therefore interpretation of all-age models should be done with caution[13,24]. However, given the high seroprevalence (60-80% among adults) and the high burden of TT experienced in these districts, it seems likely that a considerable proportion of the seropositivity observed was driven by historical trachoma transmission. Future studies that elucidate the prevalence of urogenital infections among adults in areas of trachoma persistence could be beneficial in clarifying serologic data and its potential use in programmatic decision making.

The use of serology is scaling up around the trachoma endemic world, and platforms such as the lateral flow assay are being considered to allow for serological data to be generated quickly and within the countries where it is collected [28,40]. In the meantime, it will likely remain more feasible from a programmatic sense to collect specimens from smaller age ranges, such as ages 1 to 5 years or 1 to 9 years, for which simpler logistic models for SCR may suffice. However, results of this study demonstrate the value of modeling all-age serology data in epidemiological complex settings. Collecting seroprevalence or SCR data at the district level within these important age groups allows for a better understanding of transmission patterns and may one day inform stop or start MDA programmatic decision making [24].

This study had certain limitations. Both antigens used in this study are *Ct-*specific, however as noted above, seropositivity for the antigens cannot exclude urogenital infection. This is not likely to affect the results presented on children ages 1 to 9 years, the primary subgroup monitored by trachoma programs. Time points of Pgp3 SCR change for Goncha and Debay Tilatgin were identified only in the Model 2 with added informative priors. These results differed from CT694 SCR estimates, where we found no evidence of a time point of change. These differences may be due to the nature of the antigens themselves or may be due to the fact that district level priors were not available for these districts. Informative priors were obtained from a previous study in districts with lower trachoma endemicity which may have affected the models [20]. Therefore, it is possible that the rates of seroreversion and proportional decline in transmission for Goncha and Debay may have been overestimated, which may have made the estimated time point of change less reliable. As more longitudinal serology data becomes available among districts with varying levels of seropositivity, informative priors may be improved for future studies within trachoma-persistent districts. Lastly, districts were chosen purposely from the subset of districts programmatically due for an impact survey in 2019, and therefore their selection was not random. The selected districts do represent a range of settings among districts considered persistent and therefore may be informative to endemic settings experienced by the global community.

## Conclusions

Persistent trachoma is a serious threat to reaching trachoma elimination as a public health problem by 2030, and quality epidemiological data are needed to characterize these settings. While all four of the study districts meet the definition of persistent trachoma, the TF results, bolstered by the complementary indicators, demonstrated a wide range of endemicity and programmatic needs between the districts. Districts which are persistently highly endemic such as Ebinat, would likely benefit from the implementation of enhanced interventions such as more frequent than annual MDA as soon as possible. This study also adds to the growing body of evidence that infection and serologic methods may be useful in trachoma program evaluation and decision making, particularly in settings slow to reach elimination thresholds. Further investigations of improved monitoring and enhanced interventions are required to specifically address the challenge of persistent trachoma and to achieve the elimination of trachoma as a public health problem by 2030.

## Supporting information

S1 Fig Geographic location of the four study districts, Amhara, Ethiopia, 2019. Map created in ArcGIS Pro 2.2.6 (ESRI, Redlands, CA) using shapefiles sourced from the GADM database (gadm.org).
(TIF)

S2 Fig Prevalence of TF among children ages 1-9 years, 2001-2019, among the four study districts. (DOCX)

S3 Fig Mass Drug Administration coverage for all known years of SAFE strategy intervention with antibiotic MDA, Amhara, Ethiopia. (DOCX)

S4 Fig Seroprevalence to trachoma antigens, Amhara, Ethiopia, 2019. (DOCX)

S5 Fig SCR modeling plots by district for Pgp3, Amhara, Ethiopia, 2019. (DOCX)

S6 Fig SCR modeling plots by district for CT694, Amhara, Ethiopia, 2019. (DOCX)

S1 Table Prevalence of water, sanitation, and hygiene variables within the four study districts, Amhara, Ethiopia, 2019. (DOCX)

S2 Table Complete all-age results of serocatalytic SCR modeling by antigen and district, Amhara, Ethiopia, 2019. (DOCX)
