# Supplementary figures and images for: Prevalence of ocular Chlamydia trachomatis infection and antibodies within districts persistently endemic for trachoma, Amhara, Ethiopia
Source: PLoS Negl Trop Dis. 2025 Mar 11;19(3):e0012900. doi: 10.1371/journal.pntd.0012900 (PMC11936273; doi:10.1371/journal.pntd.0012900)

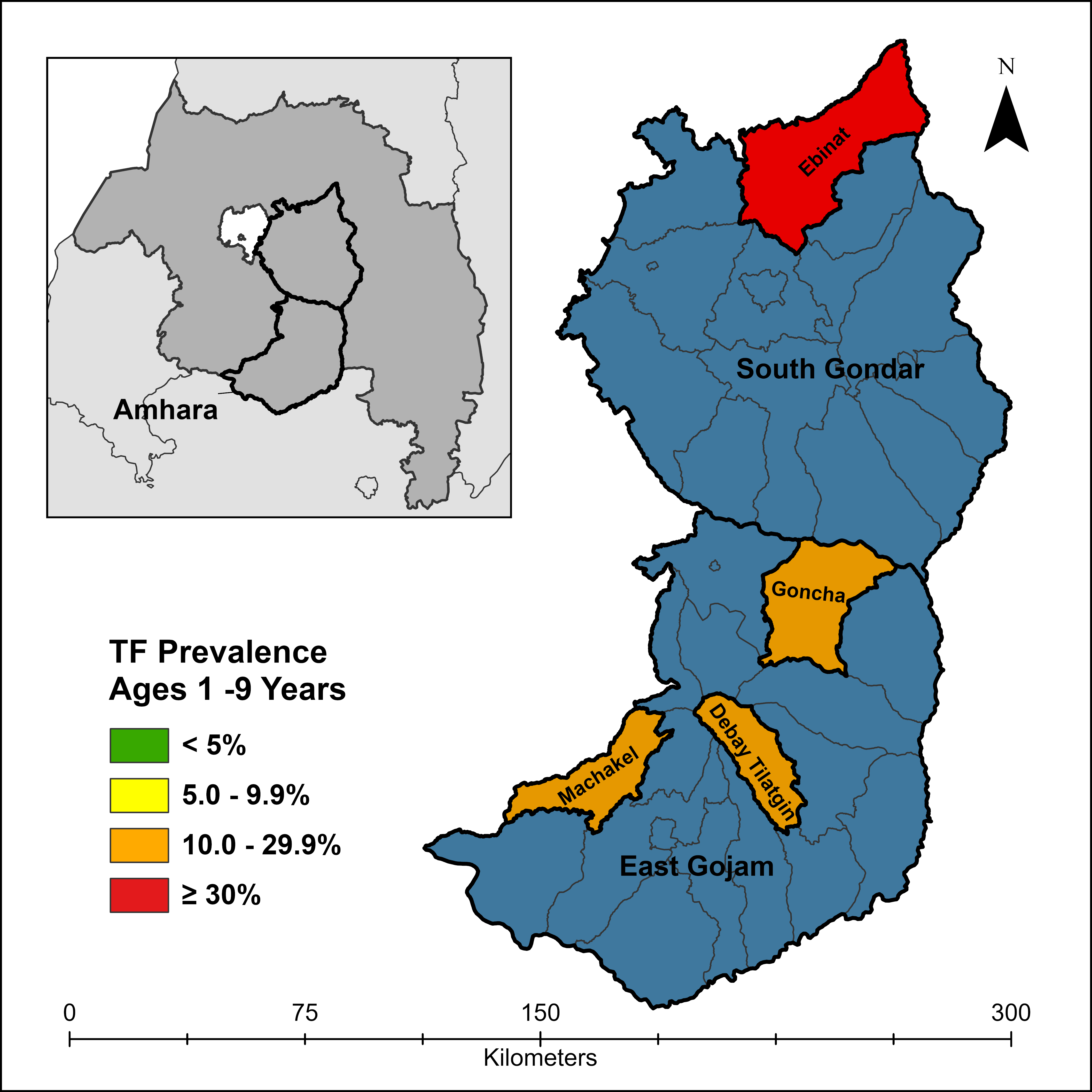

Supplement: S1 Fig — Map created in ArcGIS Pro 2.2.6 (ESRI, Redlands, CA) using shapefiles sourced from the GADM database (gadm.org). (TIF) [file pntd.0012900.s001.tif]
