# Supplementary material for: Prevalence of ocular Chlamydia trachomatis infection and antibodies within districts persistently endemic for trachoma, Amhara, Ethiopia
Source: PLoS Negl Trop Dis. 2025 Mar 11;19(3):e0012900. doi: 10.1371/journal.pntd.0012900 (PMC11936273; doi:10.1371/journal.pntd.0012900)
Supplement: S2 Fig — (DOCX) [file pntd.0012900.s002.docx]

**S2 Fig. Prevalence of TF among children ages 1-9 years, 2001-2019, among the four study districts.**


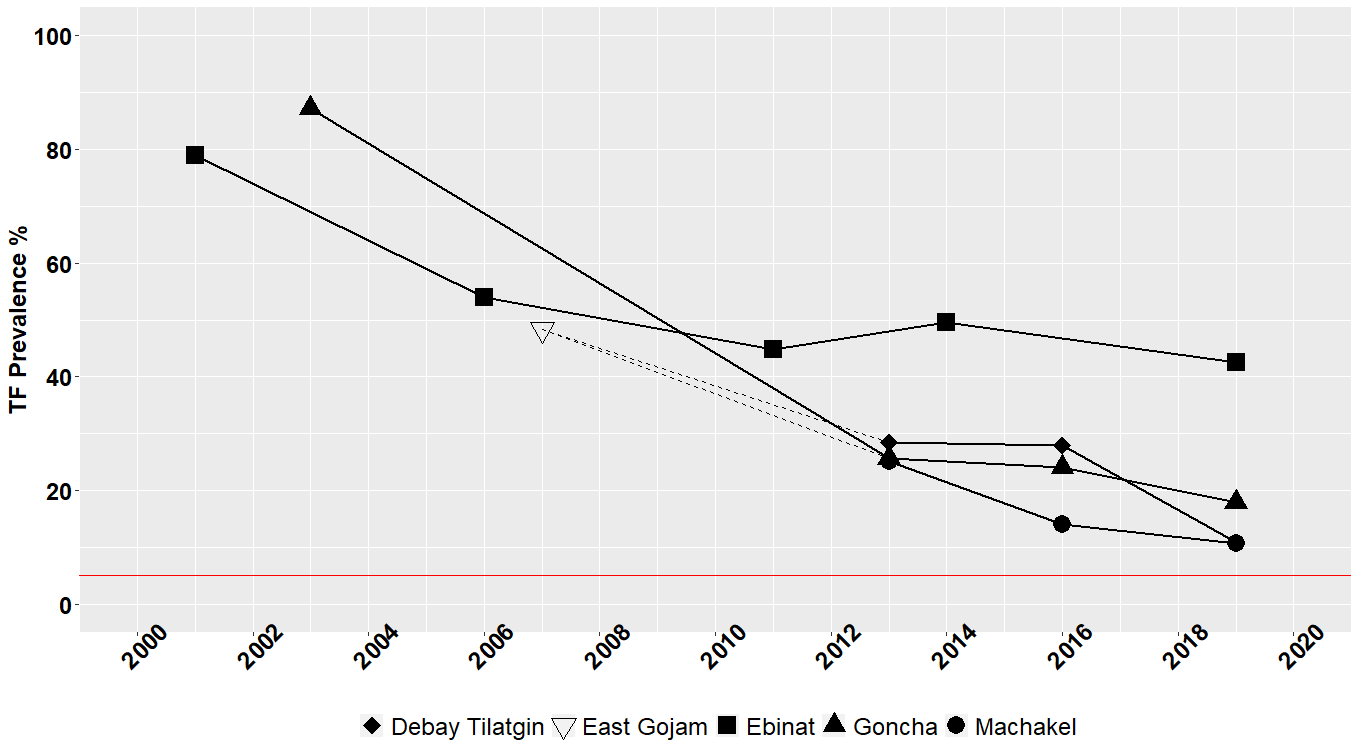


All four districts remain above the 5% elimination threshold for TF, indicated by the horizontal red line. Baseline for Ebinat and Goncha were at first time point of their respective district lines. Baseline for Machakel and Debay Tilatgin occurred at the zonal level East Gojam (EG) survey in 2007, shown by the triangle connecting to each respective line. Zonal estimates cannot be directly compared to those at the district level, and this is displayed by the dotted line.
