# Supplementary material for: Prevalence of ocular Chlamydia trachomatis infection and antibodies within districts persistently endemic for trachoma, Amhara, Ethiopia
Source: PLoS Negl Trop Dis. 2025 Mar 11;19(3):e0012900. doi: 10.1371/journal.pntd.0012900 (PMC11936273; doi:10.1371/journal.pntd.0012900)
Supplement: S3 Fig — (DOCX) [file pntd.0012900.s003.docx]

**S3 Fig. Mass Drug Administration coverage for all known years of SAFE strategy intervention with antibiotic MDA, Amhara, Ethiopia.**


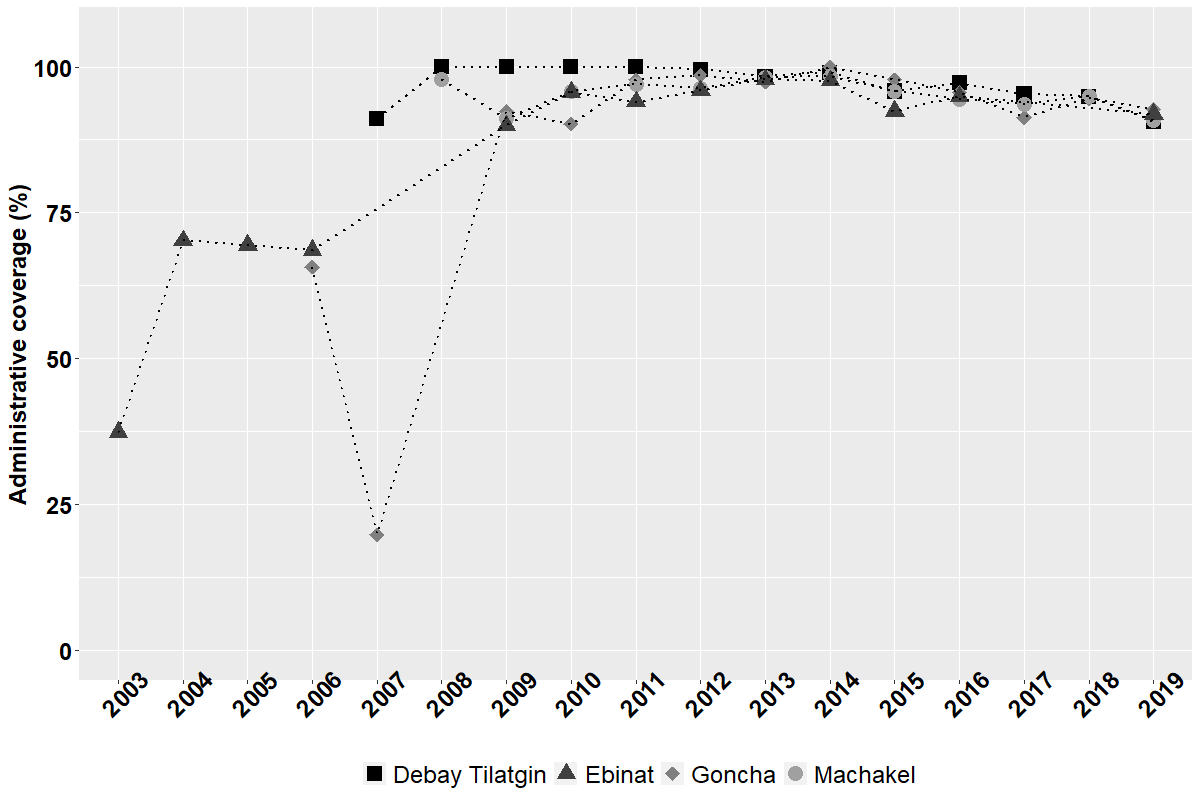


SAFE: Surgery, Antibiotics, Facial Cleanliness, and Environmental Improvement.
