# Supplementary material for: Prevalence of ocular Chlamydia trachomatis infection and antibodies within districts persistently endemic for trachoma, Amhara, Ethiopia
Source: PLoS Negl Trop Dis. 2025 Mar 11;19(3):e0012900. doi: 10.1371/journal.pntd.0012900 (PMC11936273; doi:10.1371/journal.pntd.0012900)
Supplement: S4 Fig — (DOCX) [file pntd.0012900.s004.docx]

**S4 Fig. Seroprevalence to trachoma antigens, Amhara, Ethiopia, 2019.**


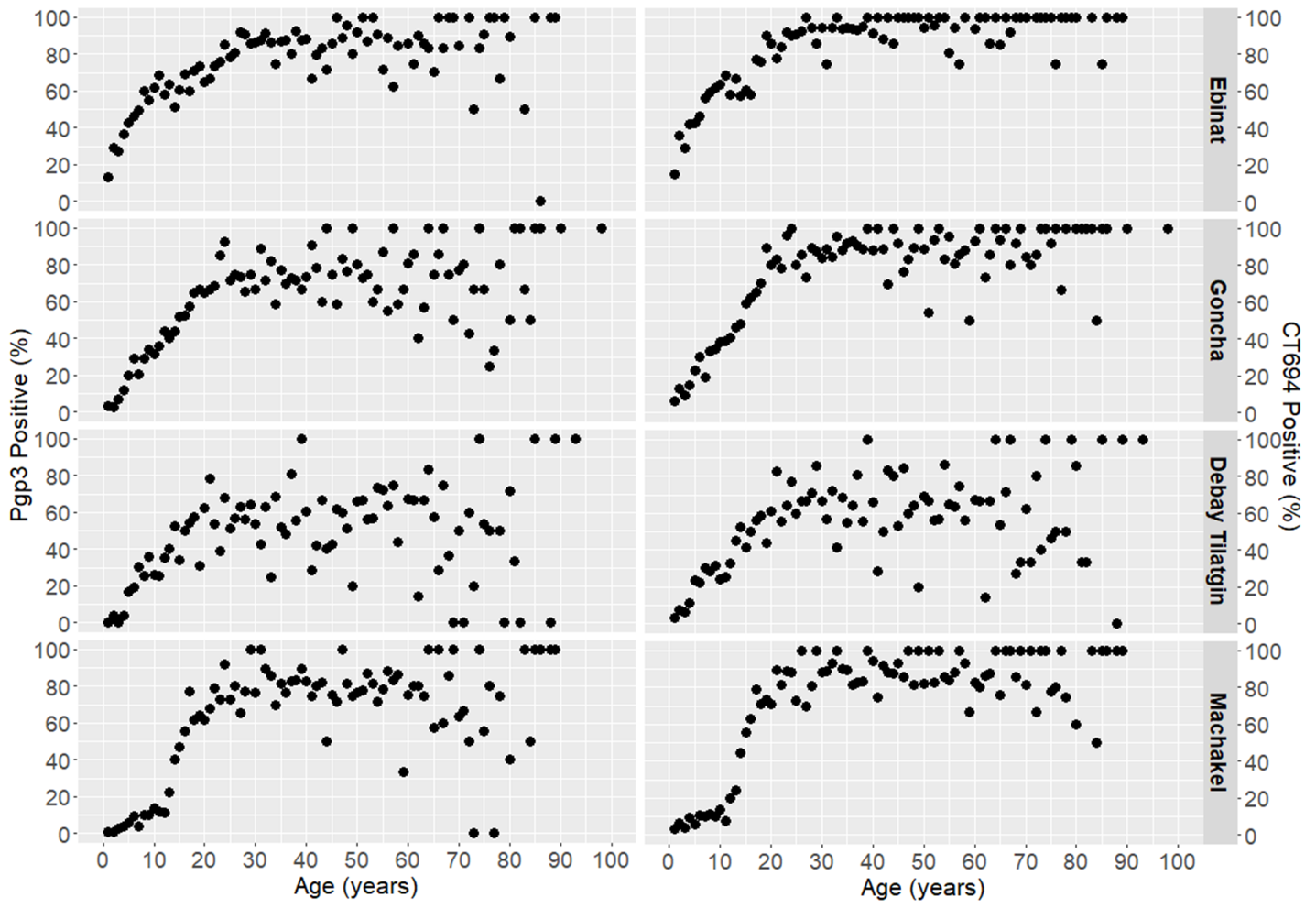


Estimated prevalence of individuals with detectable antibodies to the select trachoma antigens by year of age and district across the age range of enrolled participants.
