# Supplementary material for: Prevalence of ocular Chlamydia trachomatis infection and antibodies within districts persistently endemic for trachoma, Amhara, Ethiopia
Source: PLoS Negl Trop Dis. 2025 Mar 11;19(3):e0012900. doi: 10.1371/journal.pntd.0012900 (PMC11936273; doi:10.1371/journal.pntd.0012900)
Supplement: S5 Fig — (DOCX) [file pntd.0012900.s005.docx]

**S5 Fig. SCR modeling plots by district for Pgp3, Amhara, Ethiopia, 2019.**


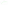


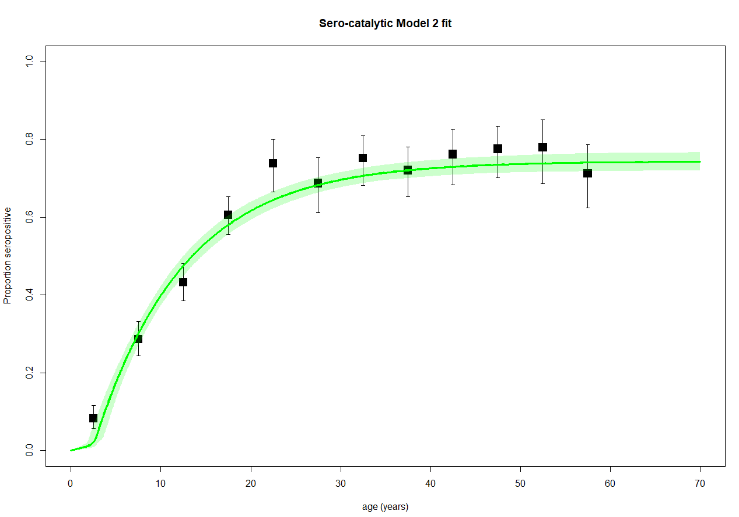

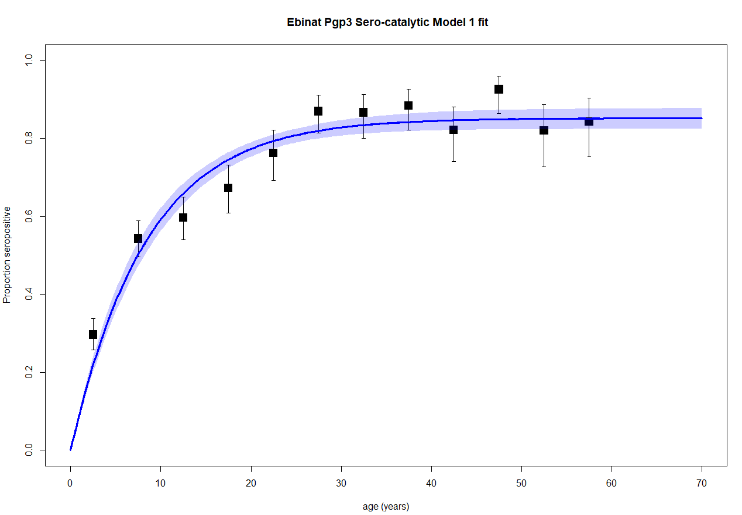


A
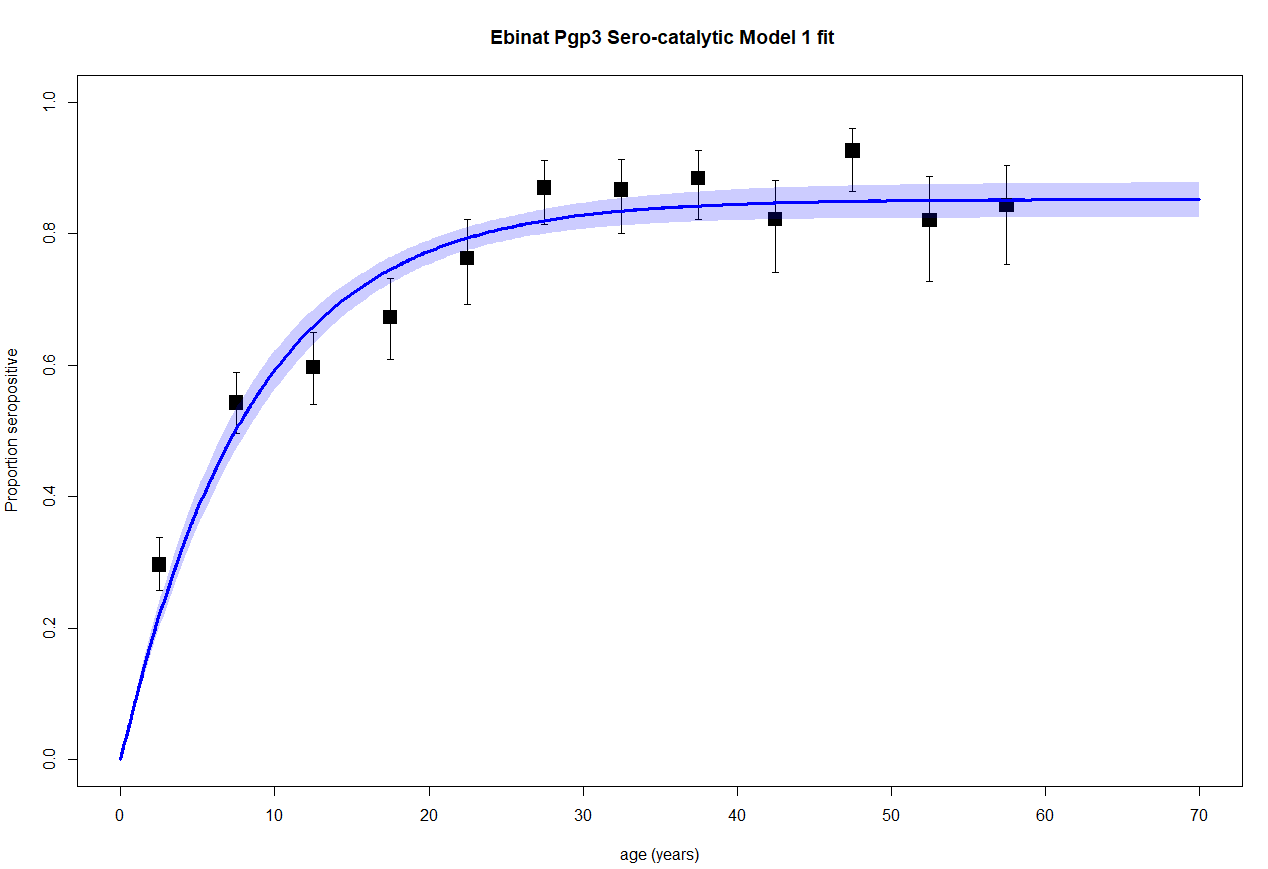


B
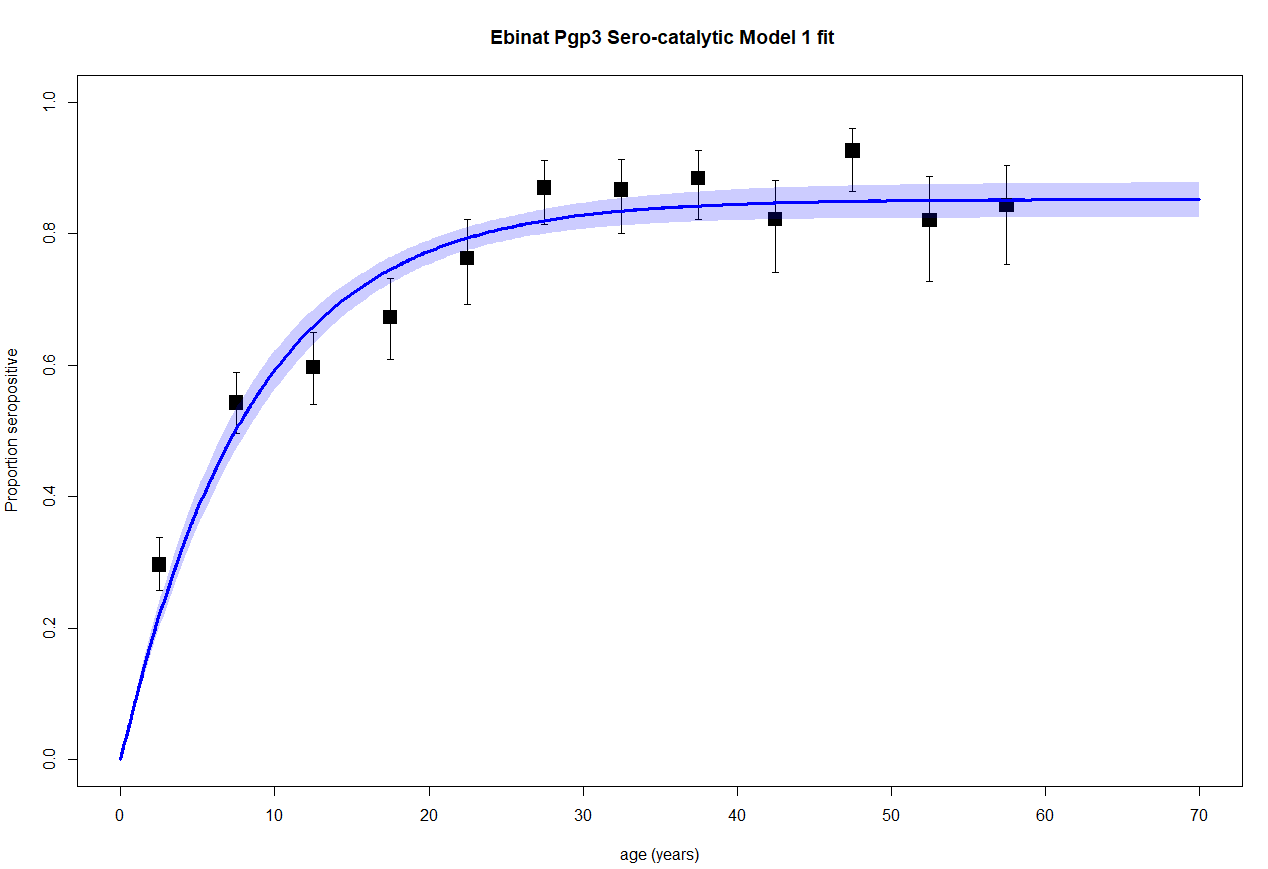


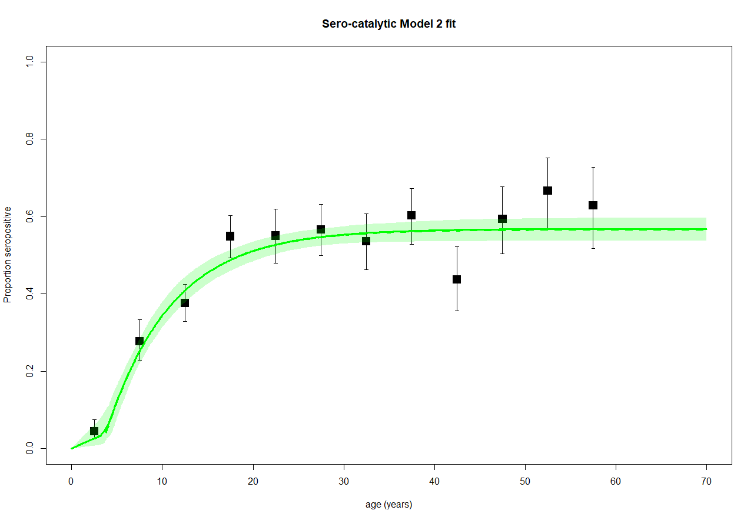


C
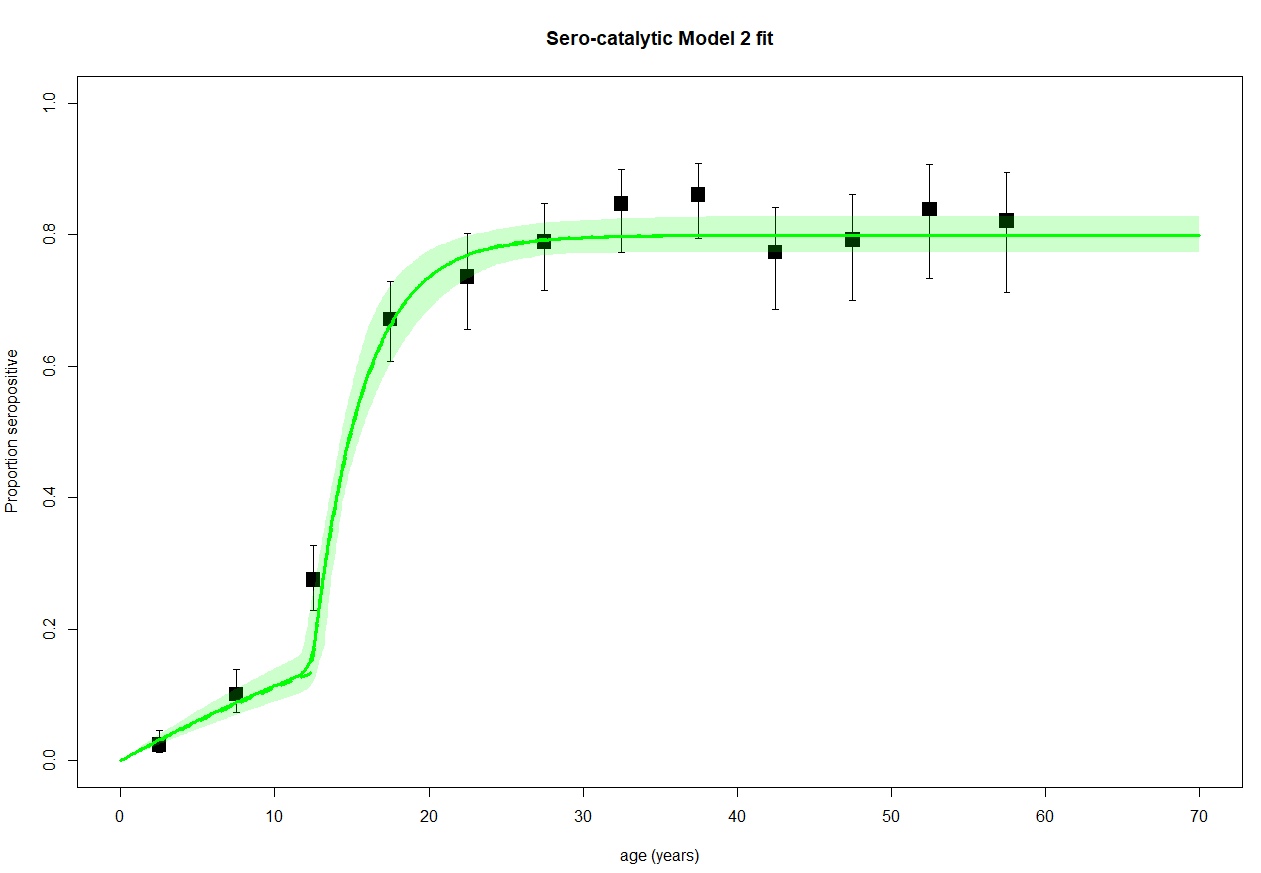

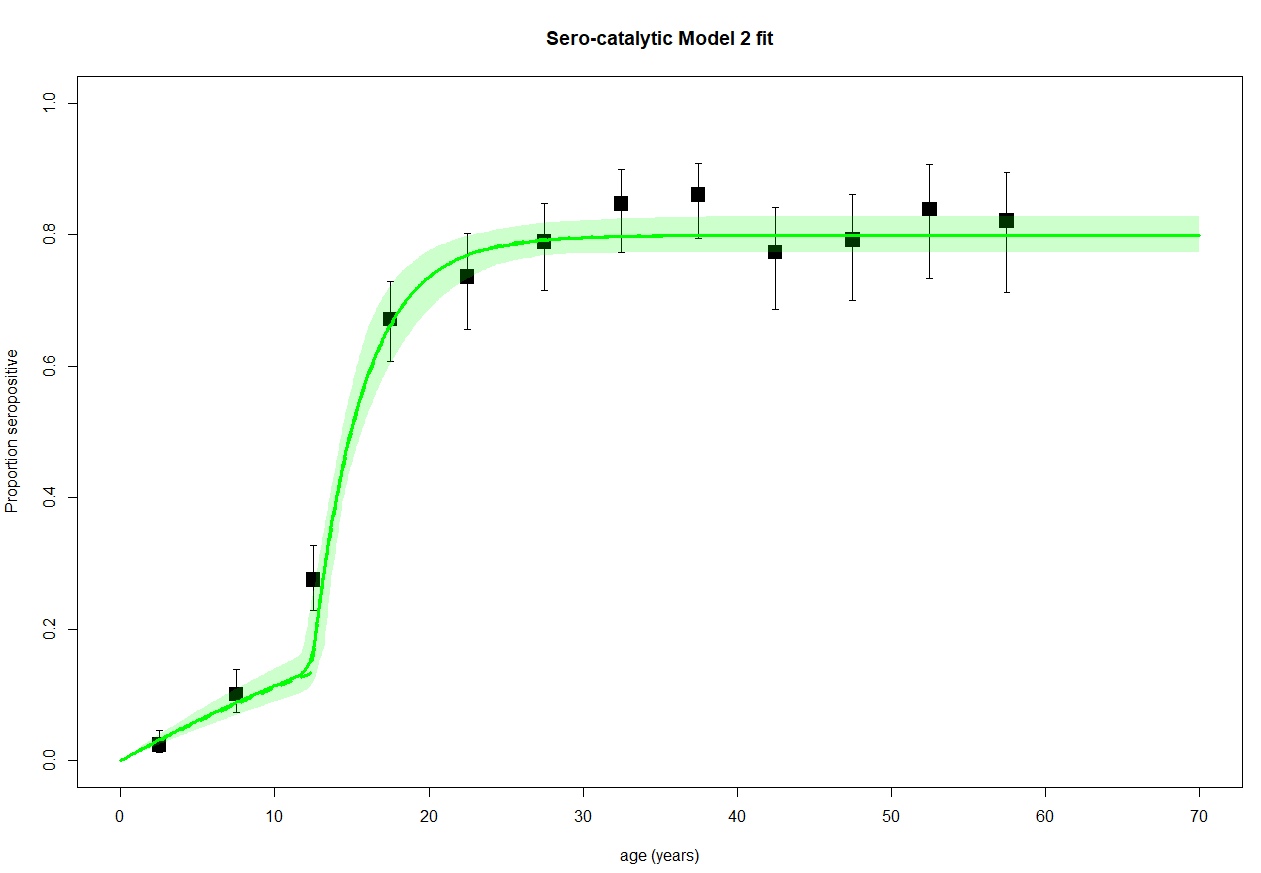

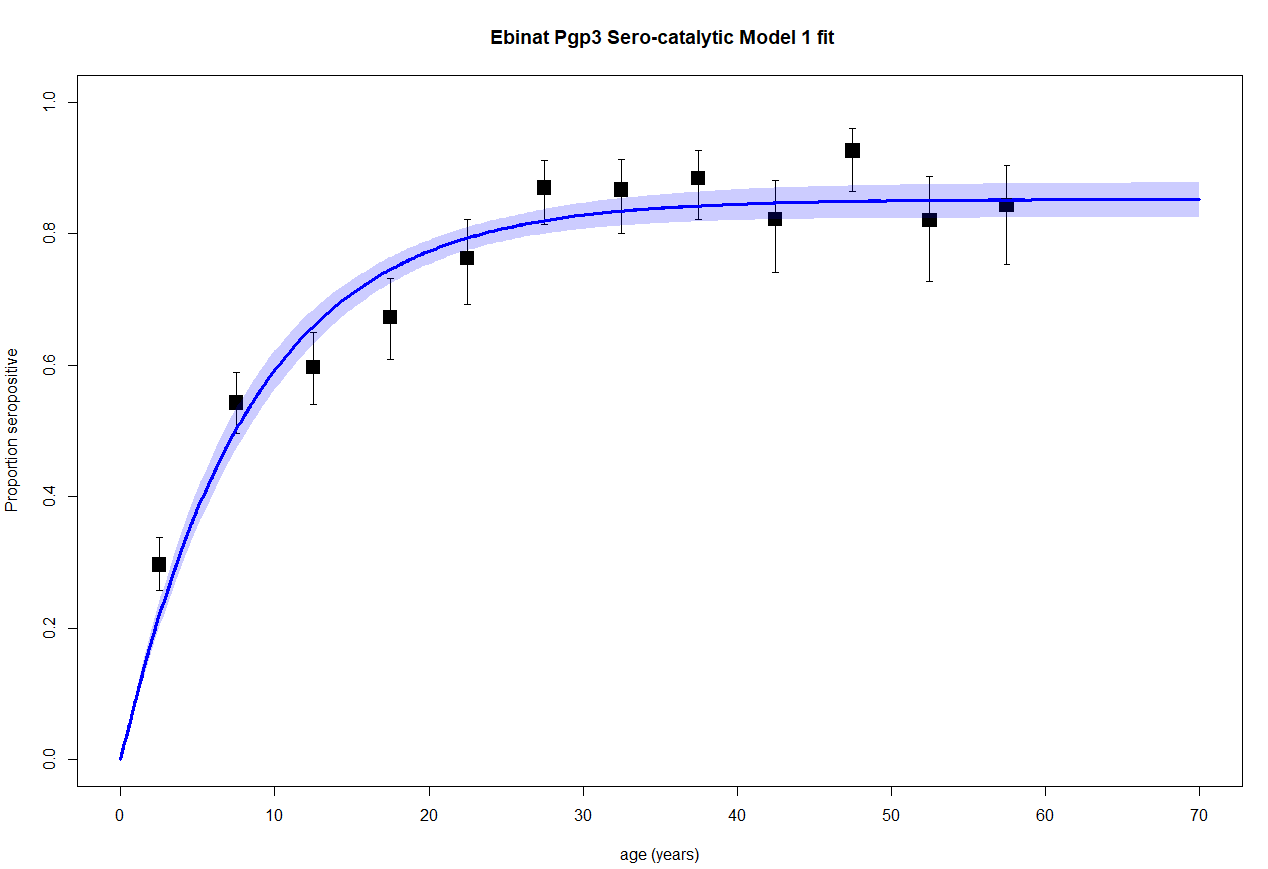


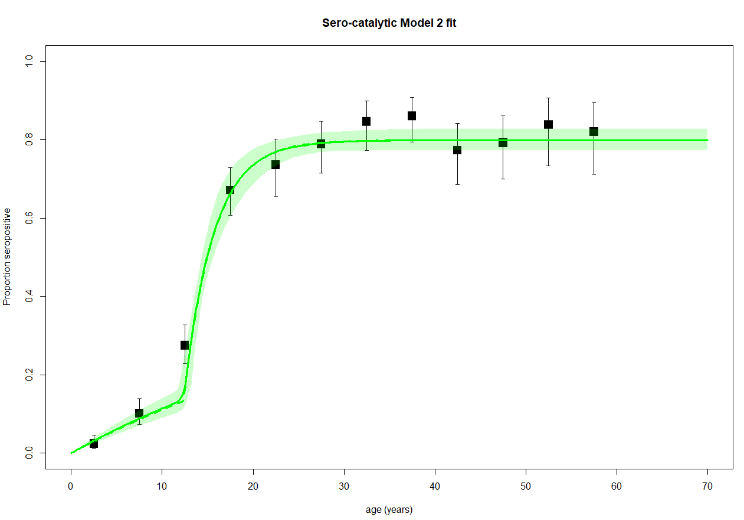


D
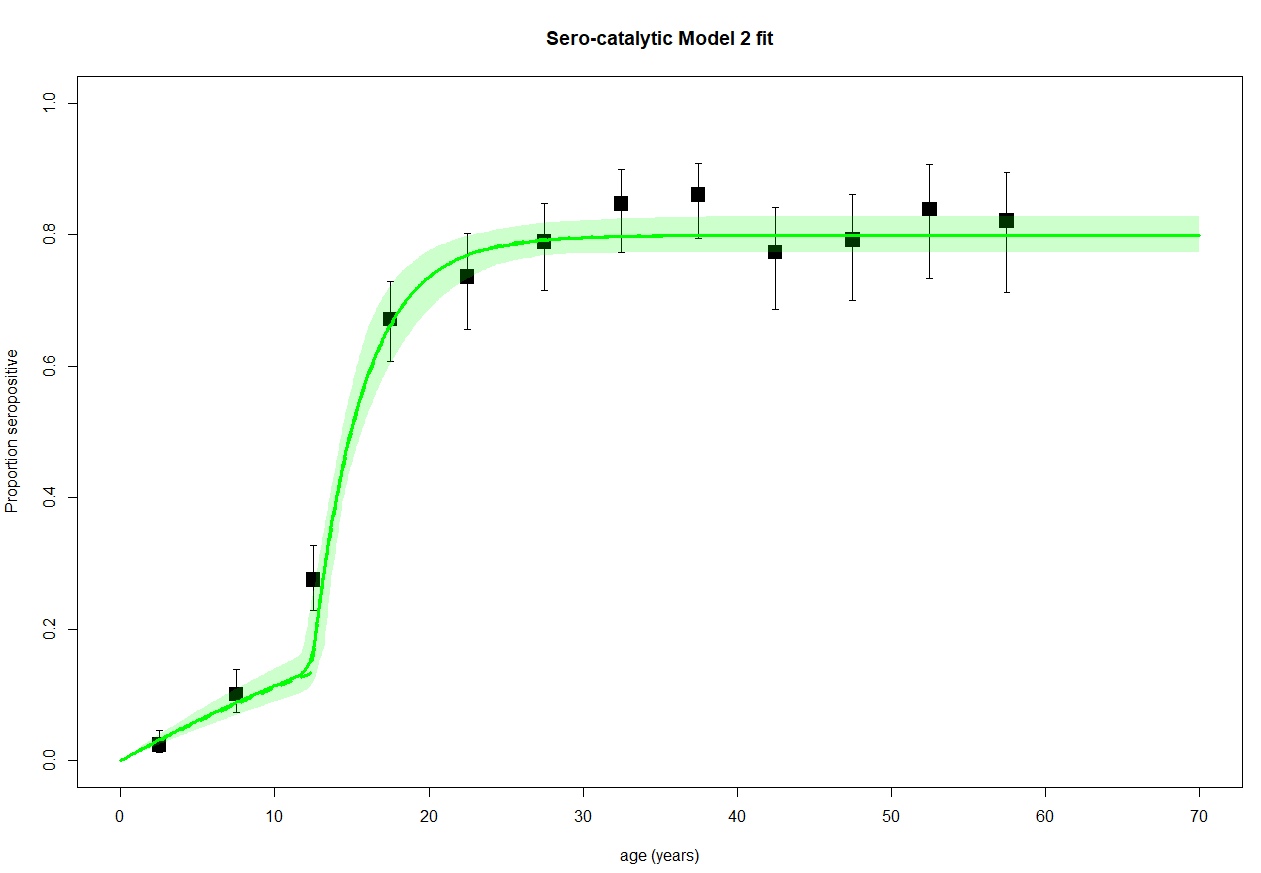

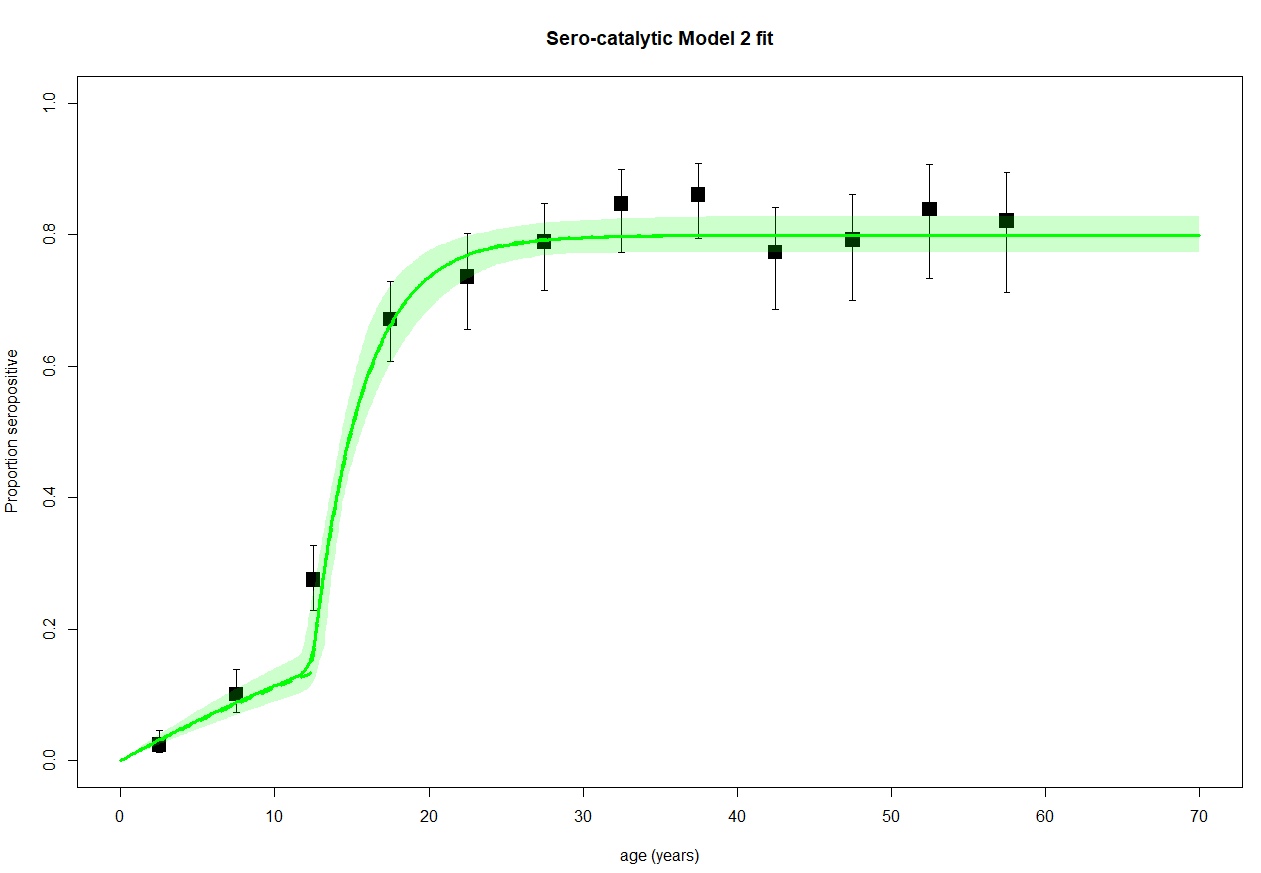

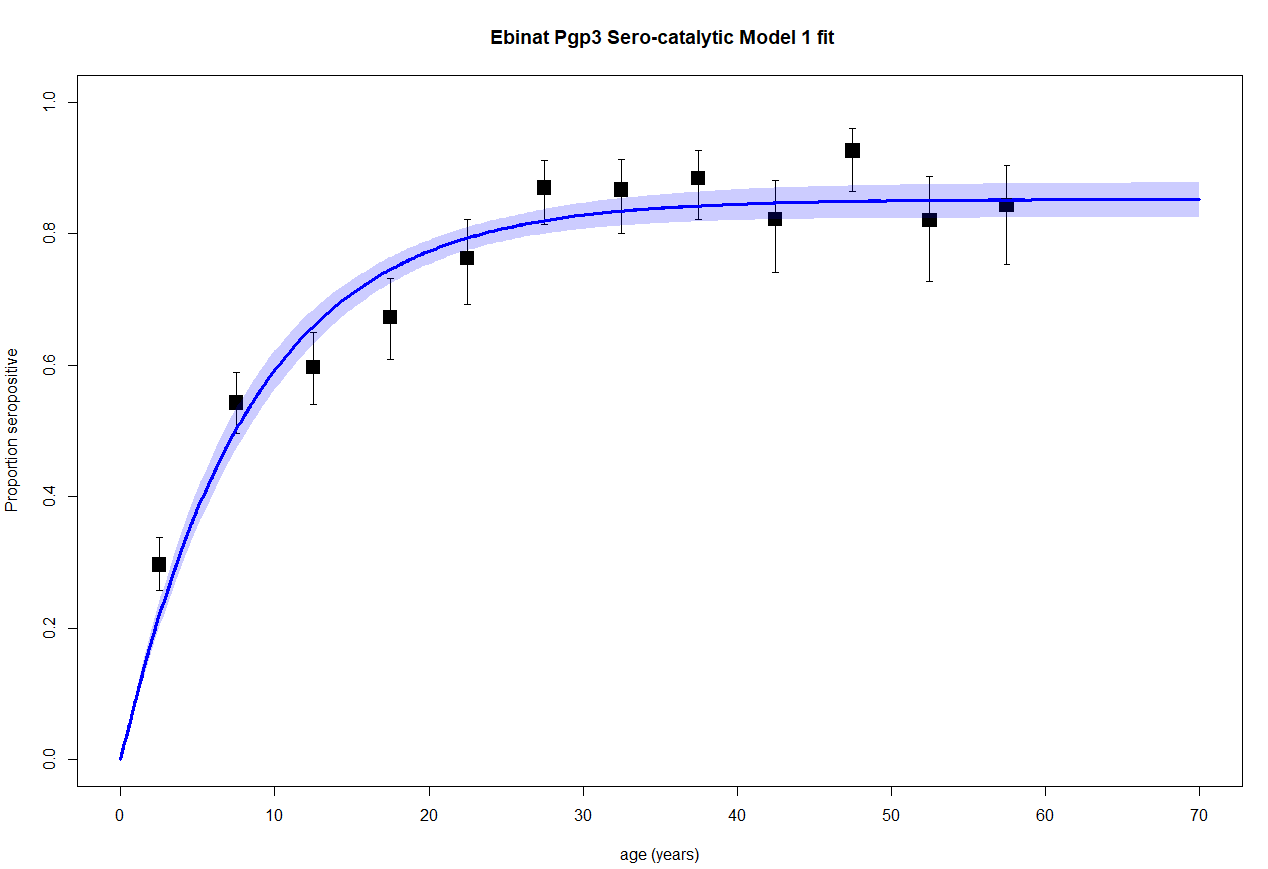


Pgp3 seroprevalence by 5-year age groups with 95% CI are shown with squares and whiskers. Overlayed in color are the estimated age-specific curves for the best fitting model with smoothing effect. Blue modeling plots represent Model 1 was best-fitting and green represent Model 2 was best fitting for the corresponding district. Upper left: Ebinat, upper right: Goncha, Lower left: Debay Tilatgin; Lower right: Machakel.
