# Supplementary material for: Prevalence of ocular Chlamydia trachomatis infection and antibodies within districts persistently endemic for trachoma, Amhara, Ethiopia
Source: PLoS Negl Trop Dis. 2025 Mar 11;19(3):e0012900. doi: 10.1371/journal.pntd.0012900 (PMC11936273; doi:10.1371/journal.pntd.0012900)
Supplement: S1 Table — (DOCX) [file pntd.0012900.s007.docx]

**S1 Table. Prevalence of water, sanitation, and hygiene variables within the four study districts, Amhara, Ethiopia, 2019.**

| **DISTRICT** | **latrine presence** | **improved latrine** | **time to water <30 minutes** | **improved water** | **clean face** |
| --- | --- | --- | --- | --- | --- |
| Ebinat | 19.0%  (16.5, 21.7) | 2.3%  (1.5, 3.6) | 30.4%  (27.4, 33.5) | 43.5%  (40.2, 46.8) | 55.1%  (50.7, 59.5) |
| Debay Tilatgin | 50.8%  (47.5, 54.1) | 1.0%  (0.5, 2.0) | 42.0%  (38.7, 45.3) | 66.0%  (62.8, 69.1) | 73.7%  (68.9, 77.9) |
| Goncha | 50.9%  (47.6, 54.2) | 0.3%  (0.1, 1.1) | 33.9%  (30.9, 37.1) | 50.4%  (47.1, 53.8) | 65.0%  (60.4, 69.4) |
| Machakel | 71.8%  (68.7, 74.7) | 0.7%  (0.3, 1.5) | 37.1%  (34.0, 40.4) | 51.2%  (47.9, 54.5) | 72.7%  (68.4, 76.5) |

95% Confidence intervals in parentheses.
