# Supplementary material for: Prevalence of ocular Chlamydia trachomatis infection and antibodies within districts persistently endemic for trachoma, Amhara, Ethiopia
Source: PLoS Negl Trop Dis. 2025 Mar 11;19(3):e0012900. doi: 10.1371/journal.pntd.0012900 (PMC11936273; doi:10.1371/journal.pntd.0012900)
Supplement: S2 Table — (DOCX) [file pntd.0012900.s008.docx]

**S2 Table. Complete all-age results of serocatalytic SCR modeling by antigen and district, Amhara, Ethiopia, 2019.**

| DISTRICT | ANTIGEN | Model | λ_0_ (95%CrI) | GR | ESS | γ (95%CrI) | GR | ESS | ρ(95%CrI) | GR | ESS | λ_1_ (95%CrI) | T_C_ | GR | ESS | LL | DIC |
| --- | --- | --- | --- | --- | --- | --- | --- | --- | --- | --- | --- | --- | --- | --- | --- | --- | --- |
| Ebinat | Pgp3 | 1* | 10.1(9.1,11.3) | 1.00 | 3697.4 | -- | -- | -- | 1.8(1.3,2.3) | 1.00 | 4897.5 | -- | -- | -- | -- | 3657.5 | 2921.1 |
|  | CT694 | 1* | 10.4(9.6,11.3) | 1.00 | 2875.1 | -- | -- | -- | 0.6(0.4,0.8) | 1.00 | 4239.9 | -- | -- | -- | -- | 2798.7 | 2469.8 |
|  | Pgp3 | 2 | 52.4(13.8,99.7) | 2.92 | 25.6 | 41.8(20.8,81.4) | 2.73 | 52.2 | 1.9(1.4,2.3) | 1.01 | 3026.0 | 10.0(7.0,11.1) | 17.1(8.8,38.6) | 1.29 | 43.6 | 1056.9 | 2864.6 |
|  | CT694 | 2 | 40.4(11.4(80.9) | 1.31 | 12.0 | 27.3(14.4,90.7) | 1.29 | 27.9 | 0.7(0.5,4.0) | 1.08 | 1489.0 | 10.3(7.5,11.2) | 21.7(10.3,41.5) | 1.32 | 39.5 | 658.3 | 2393.7 |
|  | Pgp3 | 2-ip | 10.9(9.9,12.0) | 1.01 | 601.5 | 5.4(0.8,10.9) | 1.00 | 488.5 | 2.1(2.1,2.5) | 1.04 | 388.6 | 0.6(0.1,1.2) | 0.1(0.0,0.3) | 1.00 | 1906.1 | 812.8 | 2924.8 |
|  | CT694 | 2-ip | 11.3(10.4,12.2) | 1.01 | 718.8 | 5.3(0.7,10.8) | 1.02 | 316.2 | 1.0(0.7,1.3) | 1.01 | 542.1 | 0.6(0.1,1.3) | 0.1(0.0,0.2) | 1.00 | 2052.3 | 963.8 | 2480.0 |
| Goncha | Pgp3 | 1 | 5.3(4.9,5.9) | 1.00 | 6348.6 | -- | -- | -- | 1.5(1.1,1.9) | 1.00 | 9484.6 | -- | -- | -- | -- | 5346.5 | 3322.8 |
|  | CT694 | 1* | 6.3(5.8,6.8) | 1.00 | 4769.9 | -- | -- | -- | 0.5(0.3,0.7) | 1.00 | 7885.2 | -- | -- | -- | -- | 4921.6 | 2800.1 |
|  | Pgp3 | 2 | 20.6(8.5,38.4) | 1.66 | 69.7 | 21.9(12.0,42.7) | 1.43 | 218.5 | 2.5(1.9,3.0) | 1.19 | 798.7 | 4.5(3.0,5.2) | 13.1(5.7,15.5) | 1.26 | 496.5 | 2444.9 | 3286.8 |
|  | CT694 | 2 | 23.1(16.1,35.4) | 1.06 | 947.6 | 20.9(13.9,30.3) | 1.03 | 1050.3 | 1.0(0.8,1.2) | 1.01 | 1691.7 | 4.8(4.2,5.5) | 13.7(11.9,15.4) | 1.01 | 1585.6 | 2345.0 | 2739.5 |
|  | Pgp3 | 2-ip* | 7.8(6.7,9.1) | 1.00 | 3119.8 | 7.8(3.3,13.2) | 1.00 | 3556.5 | 2.2(1.8,2.6) | 1.00 | 3307.2 | 0.6(0.2,1.1) | 2.5(1.6,3.5) | 1.00 | 4203.8 | 2977.8 | 3298.5 |
|  | CT694 | 2-ip | 57.6(34.5,105.8) | 1.73 | 117.9 | 10.4(7.2,14.8) | 2.04 | 251.4 | 1.3(1.1,1.5) | 1.06 | 721.8 | 5.1(4.4,5.8) | 15.3(13.7,16.8) | 1.71 | 660.0 | 1229.4 | 2745.3 |
| Debay Tilatgin | Pgp3 | 1 | 4.9(4.3,5.6) | 1.00 | 8632.0 | -- | -- | -- | 3.4(2.6,4.3) | 1.00 | 8450.1 | -- | -- | -- | -- | 5837.1 | 3240.5 |
|  | CT694 | 1* | 5.3(4.7,6.0) | 1.00 | 6652.0 | -- | -- | -- | 2.8(2.2,3.5) | 1.00 | 9107.4 | -- | -- | -- | -- | 5864.3 | 3234.0 |
|  | Pgp3 | 2* | 9.1(6.9,12.8) | 1.00 | 1989.2 | 12.3(3.7,29.8) | 1.00 | 3881.5 | 4.7(3.7,5.9) | 1.00 | 4279.0 | 1.1(0.3,2.7) | 3.7(2.7,5.4) | 1.00 | 1840.4 | 2686.7 | 3212.0 |
|  | CT694 | 2 | 20.5(12.4,34.8) | 1.47 | 264.9 | 21.5(13.1,36.3) | 1.22 | 511.2 | 4.1(3.4,4.9) | 1.09 | 1280.7 | 4.4(3.6,5.3) | 11.7(10.0,14.5) | 1.05 | 1917.1 | 2238.7 | 3209.3 |
|  | Pgp3 | 2-ip | 50.3(29.6,77.6) | 4.20 | 751.8 | 6.7(3.5,11.2) | 1.10 | 760.8 | 3.1(2.6,3.5) | 1.54 | 1326.1 | 3.2(2.8,3.6) | 36.0(30.1,41.0) | 12.12 | 1175.9 | 1489.5 | 3232.4 |
|  | CT694 | 2-ip | 36.4(20.1,59.6) | 3.56 | 738.7 | 7.7(3.9,13.2) | 1.33 | 729.0 | 3.3(2.9,3.7) | 3.63 | 1796.4 | 3.0(2.4,3.7) | 11.0(8.2,13.9) | 10.30 | 118.8 | 1311.8 | 3224.0 |
| Machakel | Pgp3 | 1 | 3.8(3.5,4.1) | 1.01 | 2109.7 | -- | -- | -- | 0.4(0.2,0.7) | 1.00 | 3346.2 | -- | -- | -- | -- | 1903.9 | 2777.7 |
|  | CT694 | 1 | 4.5(4.2,4.9) | 1.02 | 1303.7 | -- | -- | -- | 0.2(0.1,0.4) | 1.00 | 2698.0 | -- | -- | -- | -- | 1707.9 | 2583.7 |
|  | Pgp3 | 2* | 27.5(20.6,39.0) | 1.00 | 1555.1 | 3.9(2.6,5.7) | 1.00 | 2169.9 | 1.7(1.5,2.0) | 1.01 | 1898.6 | 1.1(0.8,1.4) | 12.3(11.7,12.8) | 1.00 | 2132.9 | 1775.7 | 2476.3 |
|  | CT694 | 2* | 27.9(20.5,38.2) | 1.00 | 1419.0 | 5.4(3.8,7.7) | 1.00 | 2045.8 | 1.0(0.8,1.2) | 1.00 | 2039.8 | 1.5(1.2,1.9) | 12.4(11.4,13.0) | 1.00 | 2071.2 | 1832.0 | 2273.0 |

SCR: seroconversion rate; *: best fitting model for each antigen for each district; λ0: estimated SCR for Model 1 and estimated SCR prior to the timepoint of change for Model 2; CrI: credible interval; γ: proportional decline in transmission over time for models 1 & 2; ρ: the seroreversion rate for models 1 & 2; λ1: SCR after the timepoint of change for Model 2; T_C_: time point of change (years ago) where significant SCR decline was detected; GR: Gelman-Rubin statistic; ESS: effective sample size; LL: log-likelihood; DIC: deviance information criteria; -ip: indicates that a previously published informative priors for γ and ρ were applied to this model.
